# Hamman Syndrome in Diabetic Ketoacidosis: Unveiling a Rare and Life-Threatening Complication

**DOI:** 10.7759/cureus.75110

**Published:** 2024-12-04

**Authors:** Bouchra Daher, Fadila Kouhen, Oussama Afandi, Soukaina Laidi, Najiba Yassine

**Affiliations:** 1 Pulmonology, Cheikh Khalifa International University Hospital, Mohammed VI University of Health Sciences (UM6SS), Casablanca, MAR; 2 Radiotherapy, Cheikh Khalifa International University Hospital, Mohammed VI University of Health Sciences (UM6SS), Casablanca, MAR; 3 Endocrinology, Diabetes and Metabolism, Cheikh Khalifa International University Hospital, Mohammed VI University of Health Sciences (UM6SS), Casablanca, MAR; 4 Pulmonology, Ibn Rochd University Hospital, Casablanca, MAR

**Keywords:** diabetic ketoacidosis, hamman syndrome, pneumomediastinum, pneumothorax, subcutaneous emphysema

## Abstract

Hamman syndrome, or spontaneous pneumomediastinum, is a rare condition characterized by the presence of free air in the mediastinum, often triggered by increased intrathoracic pressure from vomiting, coughing, or intense physical exertion. Its association with diabetic ketoacidosis (DKA) is extremely uncommon. We report a case of an 18-year-old male with poorly controlled type 1 diabetes who developed DKA complicated by pneumomediastinum, subcutaneous emphysema, and a small pneumothorax. The condition was likely precipitated by Kussmaul's respiration and severe vomiting. After treatment for DKA with insulin and fluids, the patient improved, and follow-up imaging showed complete resolution of the pneumomediastinum. This case underscores the importance of recognizing and managing this rare complication in patients with DKA.

## Introduction

Spontaneous pneumomediastinum (SPM), also known as Hamman syndrome, is a rare condition characterized by the presence of free air in the mediastinum, often accompanied by subcutaneous emphysema [1]. This phenomenon, first described by Louis Hamman in 1939, typically results from the rupture of alveolar walls, allowing air to escape into the mediastinal and subcutaneous tissues [2]. While generally triggered by factors such as intense physical exertion, coughing, vomiting, or other forms of respiratory strain, SPM is a relatively uncommon event in clinical practice [3].

A particularly unusual and poorly understood clinical scenario involves the coexistence of Hamman syndrome with diabetic ketoacidosis (DKA), a severe metabolic complication most commonly observed in patients with type 1 diabetes [4]. DKA presents with hyperglycemia, ketonemia, metabolic acidosis, and electrolyte imbalances, often precipitated by factors such as infections, insulin noncompliance, or significant physical or emotional stress [5]. The development of SPM in the DKA setting is rare. Still, it is hypothesized to result from increased intrathoracic pressure, primarily due to Kussmaul breathing and severe vomiting, which are two hallmark features of DKA [6].

This report aims to highlight this unusual association and explore the potential pathophysiological mechanisms that underlie the development of Hamman syndrome in the context of DKA. By sharing this case, we aim to raise awareness of this rare yet clinically significant combination and stress the importance of prompt diagnosis and management to prevent further complications in affected patients.

## Case presentation

An 18-year-old male with a history of type 1 diabetes, diagnosed at age eight, presented to the emergency department with a 48-hour history of uncontrollable vomiting, diffuse abdominal pain, respiratory discomfort, and progressive dyspnea. His diabetes had been poorly controlled, as indicated by a recent HbA1c of 7.4% obtained two months prior. The patient also reported additional symptoms, including myalgias, headaches, and nonspecific chest tightness, which had begun a week before admission. However, he had delayed seeking medical attention for these symptoms.

While he had never experienced diabetic ketoacidosis (DKA), he had a history of occasional hyperglycemia and episodes of hypoglycemia, often due to irregular insulin dosing or missed meals. The patient had no significant history of chronic respiratory conditions or prior lung disease. His family history was notable for autoimmune conditions, with his mother having hypothyroidism and his father having rheumatoid arthritis. The patient led a sedentary lifestyle with only occasional physical activity, and he had not been regularly monitoring his blood glucose levels, particularly during periods of stress or illness.

Upon presentation, the patient exhibited tachypnea and evident signs of dehydration, including dry mucous membranes and decreased skin turgor. Despite these signs, he remained alert and oriented throughout the examination.

Vital signs included a heart rate of 110 beats per minute, respiratory rate of 30 breaths per minute, blood pressure of 115/76 mmHg, and oxygen saturation of 92% on room air. His glycemia was above 4 g/l, and he had three acetone crosses on the urine dipstick (Table 1).

**Table 1:** Initial vital signs.

Vital sign	Measurement	Normal range
Heart rate	110	60–100 beats/min (normal), >100 bpm is tachycardia
Respiratory rate	30	12–20 breaths/min
Blood pressure	115/76	90/60 mmHg to 120/80 mmHg
Oxygen saturation	92%	95–100%
Glasgow Coma Scale (GCS)	15	15

While dehydration was clinically noted, the rest of the physical examination was unremarkable. Key laboratory findings, including blood glucose, urinary ketones, white blood cell count, and arterial blood gas (ABG) results, are outlined in Table 2, which collectively confirmed the diagnosis of diabetic ketoacidosis (DKA).

**Table 2:** Comprehensive laboratory results. BUN: blood urea nitrogen.

Parameters	Measurement	Normal range
Blood glucose	370	70–100 mg/dL (3.9–5.6 mmol/L)
Urine ketones (beta-hydroxybutyrate)	+++ (Strongly positive)	Negative
Anion gap	24	8–16 mEq/L
pH	7.25	7.35–7.45
Partial pressure of oxygen (PaO_2_)	89	35–45 mmHg
Partial pressure of carbon dioxide (PaCO_2_)	18	35–45 mmHg
Bicarbonate (HCO_3_-)	6	22–28 mmol/L
Sodium (Na)	135	135–145 mmol/L
Potassium (K)	4.5	3.5–5.0 mmol/L
Chloride (Cl)	105	98–106 mmol/L
Creatinine	1.5	0.6–1.2 mg/Dl
BUN	30	7–20 mg/dL
White blood cell count	17,000	4,000–11,000/mm³
Lactate	2.5	0.5–2.2 mmol/L
Urine output	0.5	1–2 mL/kg/h (normal)
C-reactive protein	15	0–10 mg/L
Serum osmolality	Elevated (>320)	275–295 mOsm/kg

The patient was put on a ketoacidosis control protocol consisting of rehydration, intravenous insulin, and potassium supplementation and was admitted to the intensive care unit for close monitoring.

**Figure 1:**
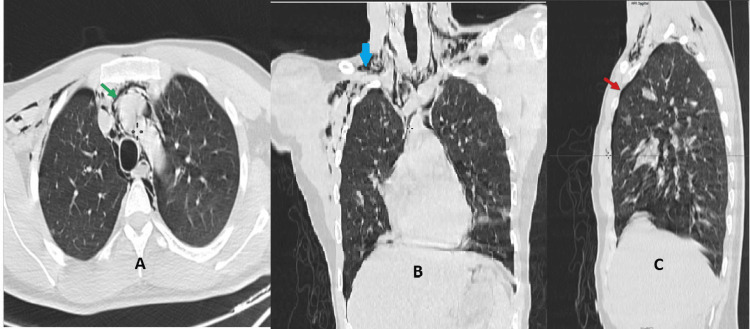
Thoracic CT imaging in axial (A), coronal (B), and sagittal (C) views showing pneumomediastinum (green arrow), subcutaneous emphysema (blue arrow), and minimal pneumothorax (red arrow).

Given the patient’s ongoing respiratory distress, he was admitted to the intensive care unit, where a contrast-enhanced thoracic CT scan was performed. The scan revealed significant pneumomediastinum, subcutaneous emphysema, and a small pneumothorax (Figure 1).

**Figure 2:**
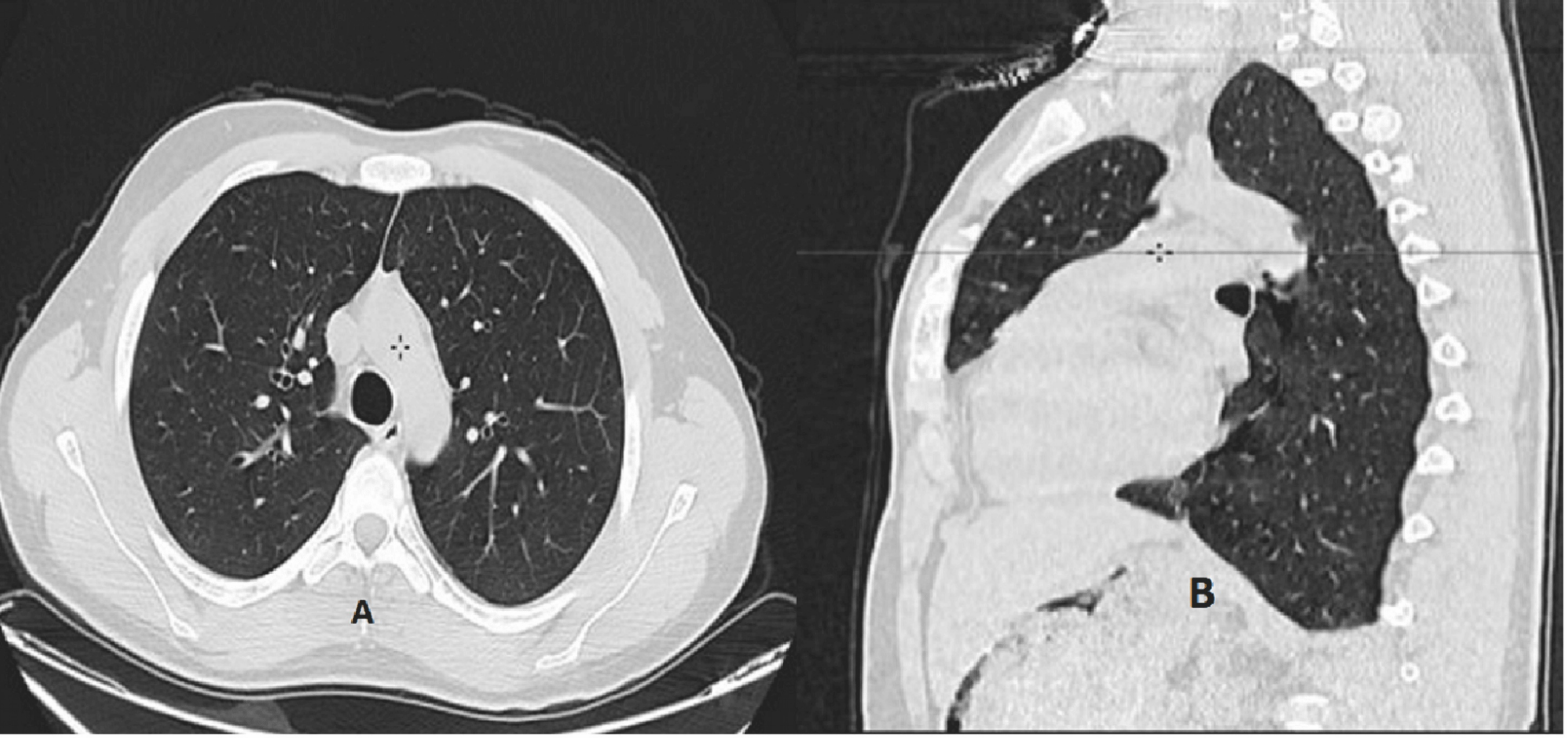
Thoracic CT imaging in axial (A) and sagittal (B) views demonstrating complete resolution of pneumothorax and pneumomediastinum.

These findings were consistent with Hamman syndrome in the context of DKA. The patient’s management involved a combination of intravenous insulin to control blood glucose levels and fluid therapy to maintain hydration and electrolyte balance. Given the respiratory distress and the findings from the thoracic CT scan, oxygen supplementation at 2 L/min was provided to support oxygenation and alleviate hypoxia. Close monitoring was instituted, with frequent assessments of vital signs, blood gas levels, and oxygen saturation to guide further management and ensure appropriate adjustments to the treatment plan. Over the next 24 hours, there was marked clinical improvement: vomiting ceased, blood glucose levels normalized, and respiratory symptoms resolved. The patient was discharged after three days in the ICU with recommendations for improved blood glucose monitoring. A follow-up CT scan performed one month later confirmed the complete resolution of pneumomediastinum, pneumothorax, and subcutaneous emphysema (Figure 2).

In terms of diabetes, the patient was put on basal-bolus insulin regimens and received therapeutic education sessions on the need for follow-up and functional insulin therapy.

To prevent the recurrence of pneumomediastinum in diabetic ketoacidosis, it is crucial to treat ketoacidosis promptly with insulin administration, adequate rehydration, and electrolyte correction. Reducing hyperventilation, often caused by acidosis, is essential by promptly correcting the acidosis and avoiding aggravating factors such as stress or pain. Identifying and treating underlying causes, such as infections or insulin omission, is also essential. Monitoring for pulmonary complications should be performed, with particular attention to avoiding efforts that increase chest pressure, such as intense coughing. Finally, patient education on diabetes management and the importance of medical follow-up is essential to prevent recurrence.

## Discussion

Spontaneous pneumomediastinum (SPM), also known as Hamman’s syndrome, is a rare condition characterized by the presence of air within the mediastinum, often following increased intrathoracic pressure [7]. It typically arises from activities such as intense coughing, vomiting, or strenuous exertion [8]. While pneumomediastinum can occur in various clinical settings, its association with diabetic ketoacidosis (DKA) remains unusual, with only over 60 reported cases since the condition was first described in 1937. The phenomenon is most commonly observed in younger individuals (average age around 20 years) with a notable male predominance (71%), which may be attributed to greater muscle mass in men, allowing for higher intrathoracic pressures during actions like vomiting or Kussmaul respiration [9].

The pathophysiology of pneumomediastinum in DKA is multifactorial, involving a combination of severe vomiting, Kussmaul breathing, and the structural changes in lung tissue associated with poorly controlled diabetes [4]. Vomiting, often induced by acidosis, and the compensatory hyperventilation (Kussmaul breathing) seen in DKA can elevate intra-abdominal and intrathoracic pressures, which elevate alveolar pressure by 20-30 mmHg above normal inspiratory levels [10]. This significant pressure rise can cause alveolar rupture, allowing air to escape into the pulmonary interstitium and track along the bronchovascular bundles into the mediastinum. Moreover, fibrotic changes in the lungs of individuals with poorly controlled diabetes may make the alveoli more prone to rupture at lower pressures [11]. This hypothesis is supported by studies suggesting that chronic hyperglycemia and poor diabetes control lead to structural lung changes, further increasing the risk of pneumomediastinum when intrathoracic pressure is elevated.

In our case, the patient exhibited multiple risk factors for pneumomediastinum, including repeated vomiting due to both hyperglycemia and acidosis, combined with Kussmaul respiration. These factors likely contributed to alveolar rupture and the subsequent development of pneumomediastinum and subcutaneous emphysema. Notably, while chest pain is a common symptom in many cases of pneumomediastinum, it was absent in our patient, as is the case in approximately 24% of individuals with DKA-associated pneumomediastinum. This lack of chest pain may be explained by the patient's altered mental status due to the severity of the metabolic derangement or by a relative insensitivity to pain.

The hallmark clinical sign of pneumomediastinum is Hamman’s crunch, synchronous crackling or popping sounds heard at the cardiac apex, caused by air movement within the mediastinum during cardiac contraction [12]. Subcutaneous emphysema, particularly in the neck and supraclavicular regions, is another common finding. Our patient demonstrated mild subcutaneous emphysema, which is consistent with previous reports of pneumomediastinum in DKA. The elevated white blood cell count observed was likely due to the inflammatory response associated with the presence of air in the mediastinum, although no infectious etiology was identified.

CT imaging is considered the gold standard for diagnosing pneumomediastinum, as it offers superior sensitivity over plain chest radiographs in detecting small amounts of air in the mediastinum, subcutaneous tissues, or pleural spaces [13]. CT imaging revealed pneumomediastinum, subcutaneous emphysema, and a small pneumothorax, all of which resolved spontaneously with conservative management. This self-limiting nature of pneumomediastinum in the context of DKA highlights the importance of supportive care in the management of such patients.

In managing DKA complicated by pneumomediastinum, the focus remains on the correction of metabolic abnormalities [14]. Insulin therapy to control hyperglycemia and acidosis is critical, and vomiting can be managed with antiemetics and prokinetic agents like metoclopramide or domperidone, which are more effective in patients with diabetic gastroparesis. In cases where vomiting persists or there is suspicion of esophageal rupture, further interventions, including nasogastric decompression or surgical consultation, may be necessary. In cases where esophageal rupture is suspected, thoracic CT, surgical consultation, and antibiotic therapy should be pursued to exclude Boerhaave syndrome [15]. 

However, pneumomediastinum in DKA is generally self-limiting and rarely requires invasive procedures, underscoring the importance of early recognition and supportive care in these patients.

## Conclusions

Although Hamman syndrome remains a rare complication of DKA, it should be considered in the differential diagnosis when patients present with respiratory distress, chest pain, or signs of air leakage into the mediastinum. Early recognition of this rare but significant complication can lead to timely interventions and favorable outcomes, as most cases resolve spontaneously with conservative management. Further studies may help elucidate the pathophysiological mechanisms linking DKA and pneumomediastinum, and it would be prudent to include this rare association in future clinical guidelines to ensure more effective management and better outcomes for affected patients.
